# Stability and *in vivo* safety of gold, titanium nitride and parylene C coatings on NdFeB magnets implanted in muscles towards a new generation of myokinetic prosthetic limbs†

**DOI:** 10.1039/d0ra07989h

**Published:** 2021-02-08

**Authors:** Veronica Iacovacci, Irene Naselli, Alice Rita Salgarella, Francesco Clemente, Leonardo Ricotti, Christian Cipriani

**Affiliations:** The BioRobotics Institute, Scuola Superiore Sant'Anna Piazza Martiri della Libertà 33 56127 Pisa Italy christian.cipriani@santannapisa.it; Department of Excellence in Robotics & AI, Scuola Superiore Sant'Anna Piazza Martiri della Libertà 33 56127 Pisa Italy; Prensilia SRL Viale Rinaldo Piaggio 32 56025 Pontedera Italy

## Abstract

Rare earth magnets are the elective choice when high magnetic field density is required and they are particularly intriguing for inclusion in implantable devices. A safe implantation of NdFeB magnets in muscles would enable the control of limb prostheses using a myokinetic interface *i.e.*, direct control of artificial limb movements by means of magnetic tracking of residual muscle contractions. However, myokinetic prosthesis control is prevented by NdFeB magnets poor biocompatibility, at present. Here we investigated three biocompatible materials as NdFeB magnet coating candidates, namely gold, titanium nitride and parylene C, which have not been analyzed in a systematic way for this purpose, so far. *In vitro* testing in a tissue-mimicking environment and upon contact with C2C12 myoblasts enabled assessment of the superiority of parylene C coated magnets in terms of corrosion prevention and lack of cytotoxicity. In addition, parylene C coated magnets implanted in rabbit muscles for 28 days confirmed, both locally and systemically, their biocompatibility, with a lack of irritation and toxicity associated with the implant. These findings pave the way towards the development of implantable devices based on permanent magnets and of a new generation of limb prostheses.

## Abstract 2

NdFeB magnets implantation in muscles could enable limb prostheses control by means of a myokinetic interface. Parylene C proved as optimal coating for corrosion resistance, *in vitro* biocompatibility and safe implantability in rabbit muscle.

## Graphical abstract

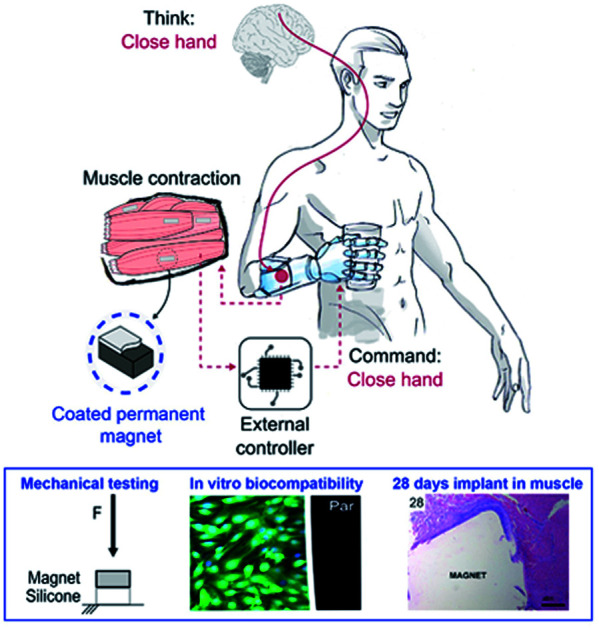

## 1. Introduction

Rare earth permanent magnets such as those made of neodymium iron boron (NdFeB) represent the elective choice when high magnetic field density is required. NdFeB magnets are feature high coercivity, remanence and volume-to-force density, thus they are appealing when a tradeoff between large magnetic field and miniaturization is required. For this reason, they have been suggested for a wide range of biomedical applications that require wireless tracking of movements and minimally invasive interventions. In particular, permanent magnets were used as key-components in several implantable devices^1^ ranging from cochlear,^2^ dental^3^ and ocular implants,^4^ to urinary^5^ and gastroesophageal sphincters^6^ and even more complex, fully implantable artificial organs.^7^ This also concerns the field of limb lengthening^8^ and prosthetics. Recently, our group proposed a new concept of human–machine interface (HMI) for the control of artificial limbs based on magnetic tracking, named myokinetic control interface.^9,10^ The idea is to use multiple permanent magnets implanted in multiple muscles in the amputated forearm to track their physical displacements during voluntary contraction. Since each magnet would travel with the muscle it is implanted in, knowledge about its position would contain a direct measure of the contraction/elongation of that muscle. This information could then be used to control proportionally the force/position of the relative movement in a limb prosthesis. More in general, by localizing multiple implanted magnets, it should be possible to monitor the contractions of the host muscles, allowing simultaneous control over multiple movements of the prosthesis, in parallel. The availability of miniaturized biocompatible permanent magnets is thus central towards the implementation of this vision, and NdFeB magnets likely represent an optimal choice, due to their excellent tradeoff between size and field density.

Biocompatibility of NdFeB magnets represents a significant issue, when direct implant is pursued. As Nd quickly reacts with oxygen, NdFeB magnets are always plated, usually with a layer of Nickel–Copper–Nickel (Ni–Cu–Ni, defined in this paper as Ni), in order to limit corrosion and prevent them from crumbling. Nonetheless, the biocompatibility of such coating, as well as its resistance to encrustations and corrosion, is extremely poor,^11,12^ thus making magnet implantation unacceptable. Despite the vast plethora of polymeric, ceramic and metallic coatings appropriate for implantable biomedical systems,^13^ just a few have been assessed as magnet coatings in order to favor a positive host response and to guarantee biocompatibility upon implantation in muscles.

Among these, titanium (Ti) presents good biocompatibility, anti-biofilm and osteogenesis activity in bone implants.^14^ NdFeB magnets encapsulated in titanium (Ti) cases demonstrated to favor their implant in ear bones,^15^ eye muscles^4^ and gastrointestinal tissues^16^,^17^ in terms of stability and biocompatibility. Nonetheless encapsulation within Ti cases significantly increases the grade of complexity of the fabrication process, as well as the invasiveness of the implantation procedure due to the case encumbrance (without increasing the volume of the magnetic material). Other examples include polytetrafluoroethylene and silicon-based coatings for subcutaneous and transcutaneous permanent magnets fixation; these were poorly assessed in terms of biocompatibility and of medium- or long-term stability.^18,19^ Good corrosion resistance was reported for titanium nitride (TiN) coated NdFeB magnets,^20,21^ although anyone assessed their *in vitro* or *in vivo* biocompatibility. Promising results were obtained with parylene C (Par) coatings. Par is a Food and Drug Administration approved material featured by almost unlimited shelf-life,^22^ high inertia and ease of deposition as a thin coating on a variety of surfaces. Nonetheless, although it was proposed for a wide range of implantable devices,^23,24^ the use of a Par coated NdFeB magnets and their biological assessment upon intramuscular implantation has been scarcely approached. The only study we are aware of is that of Fox *et al.* which proposed a magnetic attachment system for a bionic eye based on Par coated NdFeB magnets. While they evaluated its feasibility *in vivo* as well as the short term (1 day) resistance to corrosion of the magnets, they did not assess the biological host response to the implanted magnets.^25^ Hence, to the best of our knowledge, a comparative study aimed at assessing the stability and biocompatibility of NdFeB coated magnets in muscle environment, both *in vitro* and *in vivo*, also including conditions simulating muscle post-surgery inflammation, is yet to be carried out.

When selecting a coating for NdFeB magnets specific to our target application, different requirements should be taken into account to guarantee optimal performance, namely: (i) ideal coating adhesion and long term chemical and mechanical stability on the surface of the magnet; (ii) compatibility with muscle cells; (iii) moderate foreign body response/reduced fibrous tissue thickness upon implantation; (iv) resistance to corrosion also in inflammation-mimicking environments.

Here, to advance the knowledge on the matter while contributing towards the clinical implementation of the proposed HMI, we investigated three functional coatings chosen among the most promising identified by previous studies. This choice resulted from a compromise between (i) availability of preliminary data concerning the suitability of those materials in staying in contact with muscle tissues; (ii) availability of information on their processing/deposition; (iii) availability of information on the expected homogeneity of the resulting coatings. In particular we deposited (i) Au, (ii) TiN and (iii) Par on disc-shaped, general purpose, Ni coated NdFeB magnets (3 mm in diameter, 1 mm thick) and assessed their mechanical stability and resistance to corrosion in conditions simulating the target environment (*i.e.*, a contracting forearm muscle eventually in an inflammatory state). *In vitro* tests with myoblasts and *in vivo* tests on rabbits and mice were also performed, to assess the cytotoxicity, overall biocompatibility and the *in vivo* host response to the implanted magnets.

## 2. Results and discussion

### 2.1 Mechanical and corrosion resistance of the coatings

**Fig. 1:**
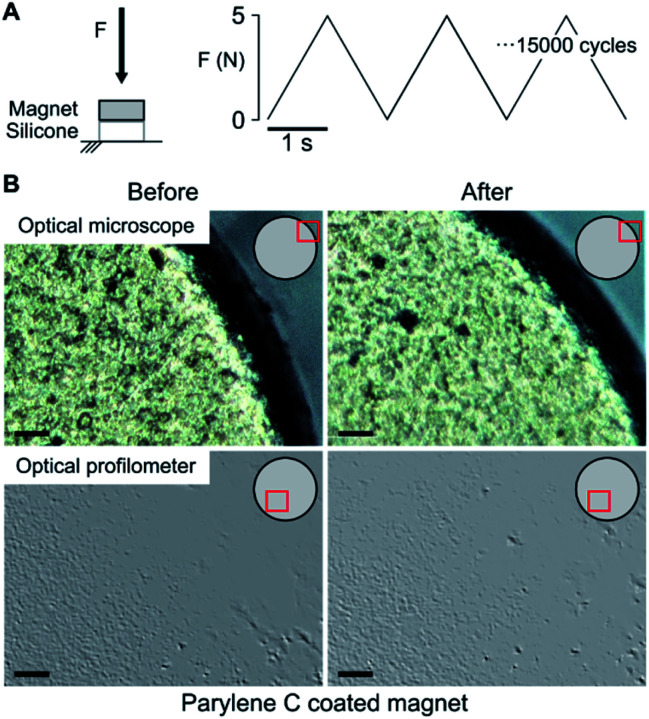
Compression stress test. (A) The magnets, standing on a silicone disc mimicking the mechanical properties of muscles, were subject to 15 000 compression cycles (5 N peak force, 0.5 Hz). (B) Visual inspection of a parylene C coated magnet using an optical microscope and an optical profilometer on a random area of the coated magnet (indicated in red on magnet schematization). No coating damages induced by the compression test could be observed.

NdFeB magnets coated with the three different materials (Au, TiN, Par) were analyzed through optical profilometry. Results (Fig. S1 and Table S1†) showed that the surface of magnets coated with gold and TiN had a comparable roughness (although with a quite different skewness), which was higher than the uncoated samples. Slightly larger roughness values were observed for Par coated magnets. Overall, these differences demonstrate that the samples were actually provided with different coatings, each one featured by characteristic surface properties. However, for all substrates the uniformity of the coating distribution and the absence of evident damages or defects was verified. This allows excluding a role of defects and inhomogeneities in the different resistance to corrosion and biocompatibility of the materials. All the magnets underwent cyclical compression loading (15 000 cycles, 0.5 Hz, 5 N compression force) in order to mimic the stress distribution in muscles and thus assess the mechanical stability of the coating (Fig. 1A). The latter is of great importance since cracks on the surface would act as trigger points for corrosion and delamination. The optical inspection of the samples (three for each coating) confirmed an optimal adhesion and stability of the three coatings as no pinholes or cracks could be observed (Fig. 1B). Since the applied stress simulated a worst-case condition (*i.e.*, an intramuscular force^26,27^ considering a ten folds corrective factor), the absence of any evident damage on the coating suggests its potential long term stability after implantation.

The resistance of the coated magnets to uniform corrosion^28^ was also assessed, in a muscle-mimicking environment which reproduced both physiological and post-surgery inflammatory conditions. Corrosion studies were performed for 30 days. This duration was chosen to verify the stability of the coating for a timeframe compatible with the planned *in vivo* test. Coated (experimental conditions) and uncoated magnets (also named Ni-magnets in the remainder of this paper; control condition) were incubated in Phosphate Buffered Saline (PBS) solution at body temperature. PBS pH was varied from 7.4 (physiological muscle) to 5 (inflammatory state) through HCl-mediated acidification. To better simulate the severe inflammatory state, the acidified PBS solution was also added with H_2_O_2_. The latter mimicked indeed the inflammatory cascade typically activated by the immune system to avoid tissue infection, and that causes the production of reactive oxidant species (ROS) and alkaline pH values around 5.

**Fig. 2:**
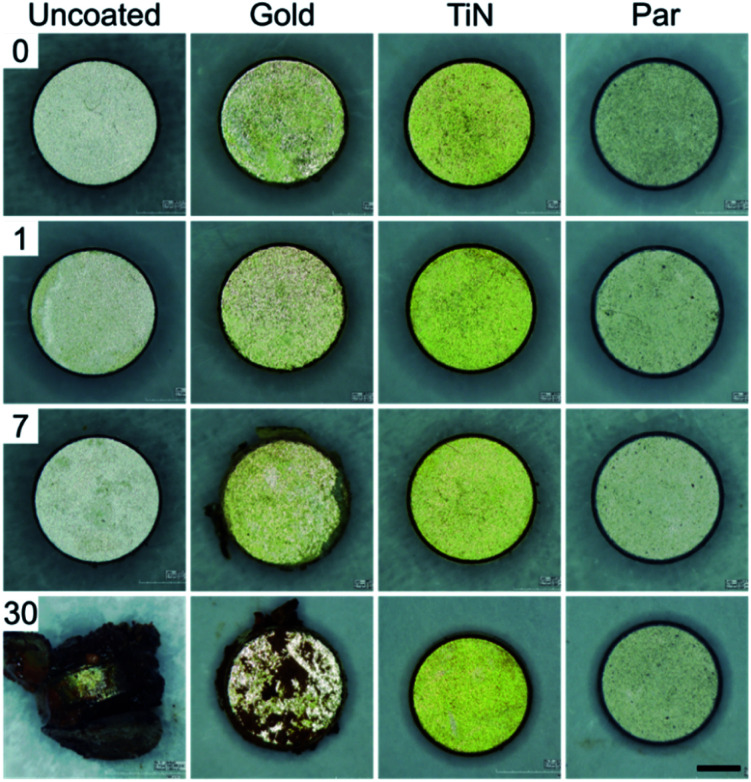
30 days corrosion test in simulated physiological muscle environment (pH = 7.4) for uncoated (control) and coated magnets (scale bar = 1 mm).

**Fig. 3:**
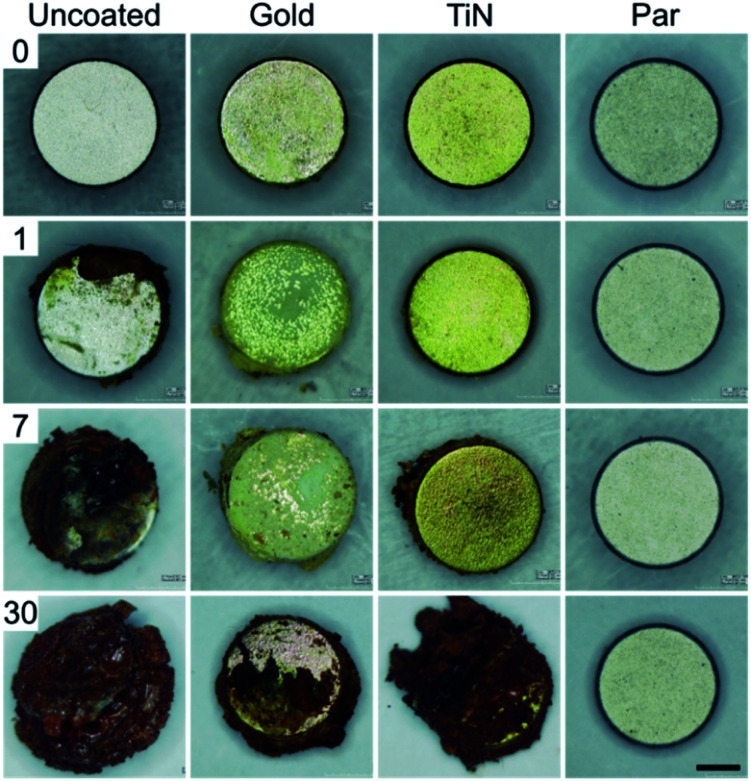
30 days corrosion test in simulated post-surgery inflammatory state environment (pH = 5) for uncoated (control) and coated magnets (scale bar = 1 mm).

Macroscopic optical inspection revealed that under physiological muscle condition (pH 7.4) the uncoated control samples started to delaminate as early as 2 weeks after immersion and ended in a complete delamination and corrosion by day 30 (Fig. 2). A thin layer of brown rust uniformly distributed over the specimen surface was found already one day after the immersion. The layer kept growing and eventually covered the surface after several days. The delamination process proved even faster in the case of immersion in the acidic environment (pH 5) with evident damages starting from day 1 (Fig. 3).

Interestingly, Au coated magnets did not react considerably better than the uncoated control samples, at both pH levels (Fig. 2 and 3). Instead, TiN and Par coated magnets demonstrated good resistance to corrosion under physiological muscle conditions, throughout the 30 days. Nonetheless, under inflammatory conditions while TiN started corrosion and oxides production from day 7 (which quickly degraded with time), Par coated magnets demonstrated excellent resistance to corrosion for the whole duration of the test (Fig. 3). These outcomes are coherent with the literature and extend the knowledge on corrosion resistance of NdFeB magnets coated with biocompatible materials.^20,29–31^ The better behavior of TiN with respect to Au^20,29^ and the remarkable inertia to corrosion of Par coated NdFeB magnets, especially with bi-layer coatings, were already reported by Noar and colleagues.^30^ However, although they assessed the exposure to saliva fluids in short term tests (1–21 days), they did not investigate the effects of muscle-like, or inflammatory-mimicking environments, thus the resistance of such coatings to acidic environments.^31^ Hence, our results confirm and expand early findings under both physiological and inflammatory muscle conditions, for a longer period. In particular, our results shed light on the effects of an inflammatory state on the stability of the implants.

### 2.2 *In vitro* biological assessment

The biological response of different magnet coatings was evaluated *in vitro* on a cellular myoblast model (C2C12 cell line), both qualitatively using fluorescence and bright field microscopy analyses, and quantitatively using WST-1 cell metabolism assay and DNA content assessment with the PicoGreen kit. C2C12 cell morphology was evaluated by imaging living cultures in direct contact with coated and uncoated magnets (control condition), in order to qualitatively assess their biological responses compared to polystyrene surfaces (used as a negative control).

**Fig. 4:**
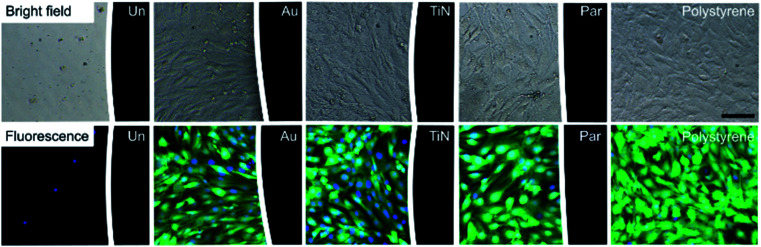
Bright field (top) and fluorescence (bottom) 10× magnification images of C2C12 cells, 72 h from seeding on polystyrene 96 well plates, in direct contact with different NdFeB coated magnets (black region on the right side of the images): uncoated (Un), gold (Au), titanium nitride (TiN) and parylene C (Par). Cells on polystyrene were used as negative control. The same trend was already observed 24 h after cell seeding (images not reported). The fluorescence images show the cytosol of viable cells in green (Calcein AM) and nuclei in blue (Hoechst). Scale bar = 100 μm.

As expected, Ni-magnets reduced cell adhesion and viability in the region adjacent to the magnet. The dramatic reduction in the number of adherent cells was well visible from the bright field image and was confirmed by staining the cells with Calcein AM and Hoechst (Fig. 4). The few adherent cells showed a pathological round shape with no esterase activity and with residual nuclei (shown in blue). These results are in accordance with the well-known Ni dose-dependent cytotoxicity that leads to cell necrosis proportionally to the metabolism inhibition grade.^32–35^

This cytotoxic effect was not observed with the coated samples. Au, TiN and Par coated magnets exhibited a good biological response without alterations of cellular adhesion, morphology and viability with respect to the cell cultured in polystyrene substrate.

**Fig. 5:**
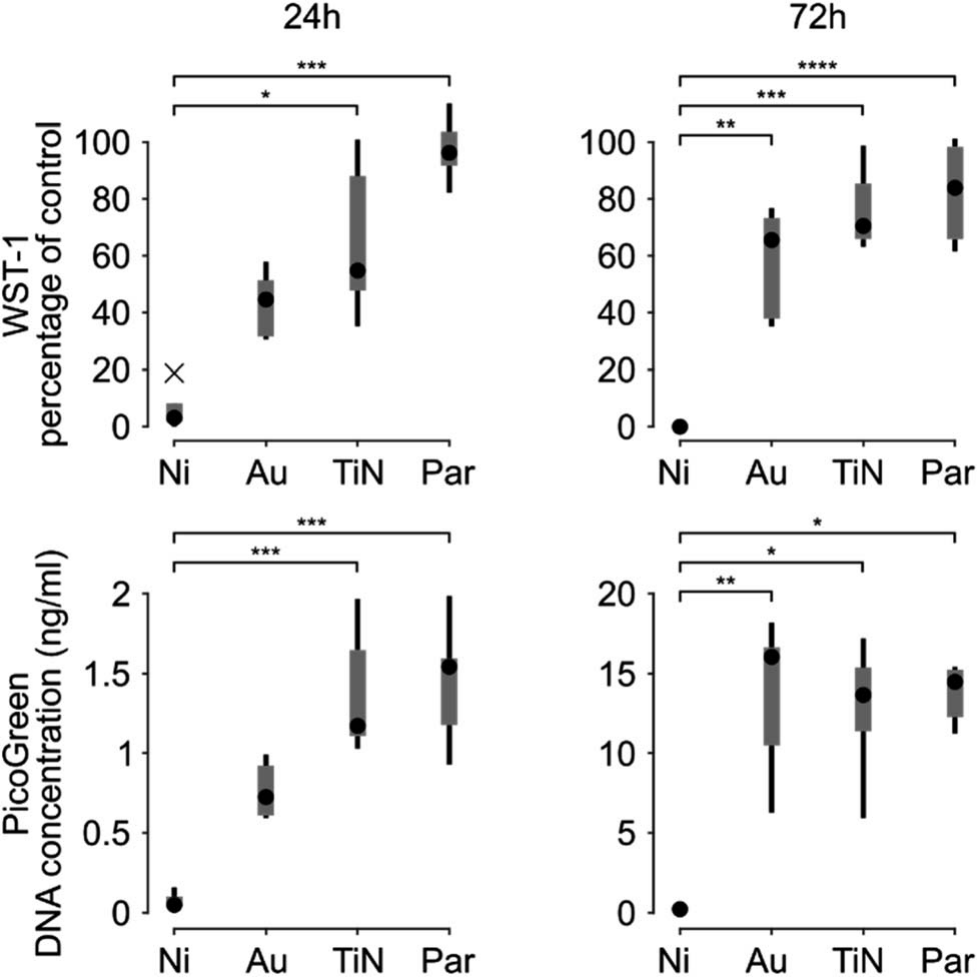
Box and whiskers plots representing the WST-1 assay results (top, expressed as the percentage of negative control and representative of cellular metabolic activity) and the PicoGreen assay (bottom, expressed as DNA concentration). Data were obtained 24 h (left) and 72 h (right) after C2C12 cell seeding (whiskers represent the maximum and minimum values of the distribution, crosses represent outliers). * = *p* < 0.05, ** = *p* < 0.01, *** = *p* < 0.001, **** = *p* < 0.0001.

A more detailed *in vitro* analysis was performed by evaluating cellular metabolic activity through WST-1 assay and DNA content by PicoGreen assay. The cytotoxicity induced by uncoated magnets was confirmed by the absence of cellular metabolic activity and DNA content, which is a direct indication of the number of adherent cells (Fig. 5).

This behavior was observed both 24 and 72 hours after seeding. The metabolic activity and the DNA content of C2C12 cells in contact with Au coated magnets proved 44.59% (median value, IQR = 51.63–31.29) of the cells on polystyrene and 0.72 ng mL^−1^ (IQR = 0.9835–0.6015), respectively, 24 h after cell seeding. These did not significantly differ from the uncoated magnets (*p* > 0.05; Kruskal–Wallis post-hoc test with Dunn's multiple comparisons). Based on evidence from the state of the art, uncoated magnets cytotoxicity may derive from Ni ions leaching^32–35^ even if Ni ions release was not quantified in this study. 72 h after seeding, instead, both the metabolic activity (*p* = 0.0024) and the number of cells in contact with Au coated magnets (*p* = 0.0025), proved significantly higher than uncoated magnets. These results are in line with previous studies on gold coatings tested with the C2C12 cell line,^36^ which showed an improved cellular proliferation with respect to an uncoated substrate (namely PDMS). However, myoblasts cultured in direct contact with Au coated magnets still exhibited a reduced metabolism with respect to polystyrene negative control (median value 65.52%, IQR = 73.56–36.94). In line with these results, Au coated magnets implanted in chickens' eyes (to monitor their position) have raised rare instances of bio-incompatibility related to damages in the gold plating.^37^

TiN coating showed better performance than Au, both in terms of metabolic activity and DNA content, and proved statistically different than uncoated magnets, 24 and 72 hours after seeding. Nonetheless, the metabolic activity remained lower compared to the negative control (median value 54.76% (IQR = 91.33–45.76), 24 h after culture and 70.47% (IQR = 92.12–64.50) after 72 h). Surprisingly these results disagree with previous evidences obtained on L929 mouse fibroblasts, where the metabolic activity reached 100% of the control two days after a culture with extracts from TiN coated samples.^38^ This discrepancy could be due to different factors: a different cell model tested (C2C12 *vs.* L929), a different deposition method used (chemical vapour deposition *vs.* physical vapour deposition), a different substrate (Ni-magnets *vs.* stainless steel) and also a different experimental strategy (direct contact *vs.* extraction).

Par coated magnets showed excellent metabolic activity and DNA content, which proved statistically different compared to uncoated magnets. The metabolic activity, proved well above 70% of the negative control (median values: 96.22% (IQR = 104.8–90.74) after 24 h and 83.90% (IQR = 99.98–63.73) after 72 h), *i.e.*, the well-known limit of cytotoxicity identified by the ISO 10993-Part 5:2009. The latter is the gold reference for the biological evaluation of medical devices in terms of *in vitro* testing of cytotoxicity. Thus, Par can be considered a safe coating.

Our findings are in line with those obtained by Bondemark *et al.*,^12^ which demonstrated that Par coated NdFeB magnets did not show cytotoxicity. It should be noted though that Bondemark *et al.* tested Par coated magnets on L929 mouse fibroblasts using two indirect cell-material contact methods, namely, the millipore filter method and the extraction method. Here instead we used the C2C12 myoblast model, which is arguably more appropriate for the foreseen application, and tested the samples in direct contact with the cells, *i.e.*, a method known to reveal both possible toxic material effects as well as the influence of the physicochemical nature of the substrate.^11^ The relevance of physicochemical material properties in biological evaluation was recently emphasized in the 2018 standard revision of ISO 10993-Part 1:2018, thus furtherly confirming the significance of our findings.

### 2.3 *In vivo* toxicity tests

Parylene C proved as the most appropriate coating for the target application, according to the results achieved in the mechanical stability, corrosion and *in vitro* cytotoxicity tests. Hence, Par only was tested *in vivo*, in order to minimize the number of exposed animals.

Acute systemic toxicity induced by Par coated magnets was evaluated in Swiss Albino Mice (*n* = 20, randomly divided in four groups) following ISO 10993-11:2017 (E). In particular, we compared the effects produced by intravenous or intraperitoneal injections of Par coated magnet extracts (in polar solvents – G2; in non-polar solvents – G4) and bare solvents (polar solvent – G1; non-polar solvent – G3). None of the four treatments produced clinical signs of toxicity or mortality (Table S2†), as well as no changes in body weight (Table S3†). No gross pathological changes were observed in any of the four groups (Table S4†), thus suggesting the overall safety of Par magnets.

Material pirogenicity and sub-acute toxicity up to 28 days post implant were assessed in New Zealand White Rabbits (following ISO 10993-11: 2017 (E) and ISO 10993-6: 2016 (E)). None of the rabbits (*n* = 6) showed an increase in temperature equal to or higher than 0.5 °C with respect to the control temperature. The average rise in temperature proved 0.167 ± 0.063 °C in the control group (*n* = 3) and 0.167 ± 0.064 °C in the animals administered with the Par coated NdFeB magnets extracts (*n* = 3) (Table S5†). Thus, no signs of pirogenicity induced by Par coated magnets were detected.

Sub-acute toxicity upon paravertebral muscle implantation (*n* = 18, divided into three groups) was evaluated. Host response induced by Par coated magnets (group GR2, *n* = 6) was compared with that of negative control samples (high-density polyethylene film, HDPE-RM-C, group GR1, *n* = 6) over a 28 days period. An intermediate assessment was performed 7 days after the surgery in rabbits implanted with both Par coated magnets and HDPE-RM-C (GR3, *n* = 6). The implantation of Par coated magnets did not produce any abnormal clinical signs, weight losses, reductions in food consumption or macroscopic alterations. At the same time, no hematological and metabolic alteration could be detected through blood and urine exams (Tables S6 and S7†).

**Fig. 6:**
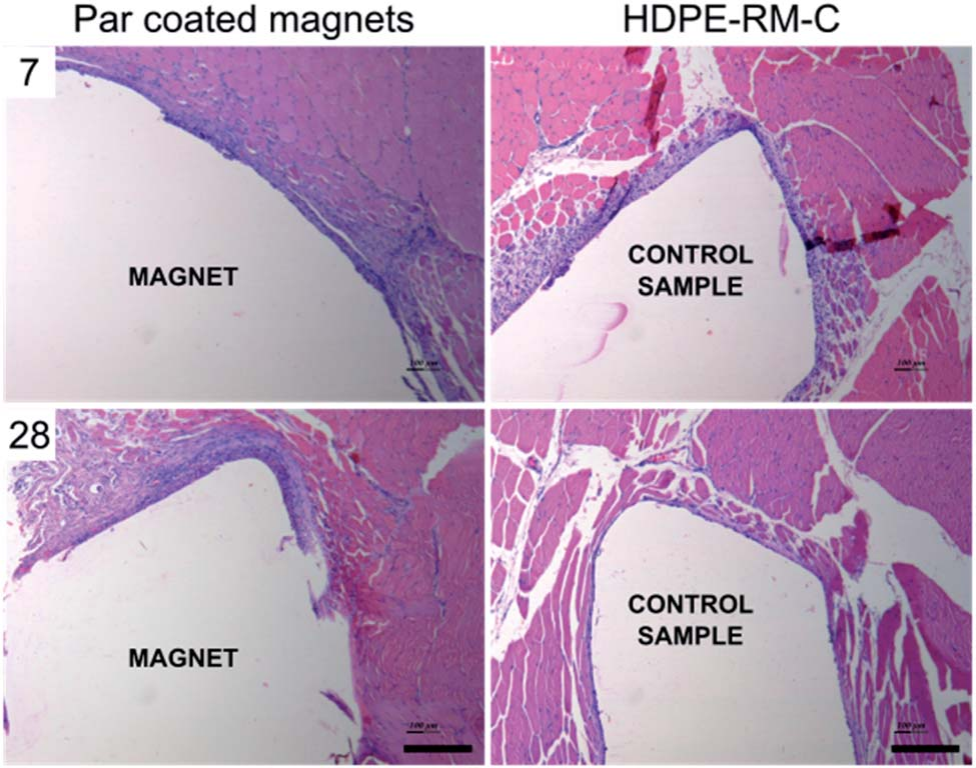
Histological examination upon paravertebral muscle implantation of parylene C coated magnets (left) and biocompatible high-density polyethylene films (HDPE-RM-C) used as negative control (right) after 7 (above) and 28 days (below) from implantation. Microscopic analysis upon paravertebral muscle implantation showed the presence of fibrous tissue, neovascularization and lymphocytes in the peri-implant area. Scale bar = 200 μm.

Microscopic observation of the implant site, 7 and 28 days after the implant enabled to evaluate the extent of the inflammatory response at two different time points (Fig. 6). At day 7, a narrow to thick band of fibrous tissue was observed at the peri-implant area, which proved slightly ticker compared to the negative control that showed a narrow to moderately thick band of fibrous tissue. Minimal neovascularization was observed in both cases. On the other hand, minimal to packed infiltration of lymphocytes was observed upon Par coated magnets implantation, as a sign of inception of the chronic inflammation.^39^ The inflammatory response of the implanted magnets proved slightly stronger compared to the negative control group, showing minimal to heavy lymphocytes infiltration.

A similar trend was observed 28 days after implantation: minimal to heavy infiltration of lymphocytes was observed in Par coated implanted animals (GR2). On the other hand, implantation sites from the negative control group (GR1) showed minimal to mild lymphocytes infiltration. Very few giant cells were observed and no tissue necrosis was identified in any of the tissue sections examined, thus witnessing no severe foreign body response.

The average width of the fibrous capsule and the average grade allocated for lymphocyte and macrophage infiltration in association with the intramuscular implantation of Par coated magnets at 7 and 28 days are reported in Table 1. Statistical analyses revealed no significant differences with respect to the control samples (*t*-test) confirming the eligibility of Par coated magnets as safely implantable systems capable of preventing severe foreign body response in the host.

**Table 1:** Microscopic observation of the implant site 7 and 28 days after the implant and comparison between parylene C coated magnets and biocompatible high-density polyethylene films (HDPE-RM-C) used as negative control

	Day 7		Day 28	
	Par coated magnet (*n* = 6)	HDPE-RM-C (*n* = 6)	Par coated magnet (*n* = 6)	HDPE-RM-C (*n* = 6)
Polymorphonuclear	0	0	2	0
Lymphocytes	68	58	44	32
Plasma cells	0	0	0	0
Macrophages	0	0	0	0
Giant cells	0	0	0	0
Necrosis	0	0	0	0
Neovascularization	12	12	13	12
Fibrosis	28	22	19	14
Fatty infiltrate	1	2	5	2
**Total**	**109**	**94**	**83**	**83**
**Irritation score**	**1.25**		**1.92**	

In order to determine the tissue response to the implant, we calculated an average score for the control (GR1) and experimental groups (GR2 and GR3). The average score from the control group was subtracted from the average score of the experimental group to derive the irritation score. This latter proved 1.25 at day 7 and 1.92 at day 28. Referring to the ISO 10993-6:2007, irritation scores in the range 0–2.9 can be considered as non-irritant. Thus, Par coated NdFeB magnets can be considered, once again, suitable for the target application and for future *in vivo* employment upon implantation in muscles.

While several evidences supporting the use of parylene C as a coating for implantable systems like animal transponders,^40^ ocular implants^25^ and subcutaneous insulin pumps^41^ were available, very little was known about the biocompatibility of this material as a coating for NdFeB magnets. The results reported in this paper extend indeed the range of safe application of parylene C to deep muscle tissues. An asset of this study is that the tests were carried out by carefully following the medical device safety ISO, by evaluating also the toxicity induced by injections of sample extracts so as to determine the biological reactivity and the hazard potential of any substances that may be released from the device into the surrounding tissues. The low irritation score associated with the Par coated magnets paves the way towards their use as a key element in a safe myokinetic implant,^9^ as well as in other biomedical applications in which a safe acute or chronic interaction of magnets with muscle tissues may be desirable,^42–44^ with possible impacts also in the field of muscle-based bio-hybrid robotics.^45^

## 3. Conclusions

NdFeB magnets are extremely appealing as implantable systems or part of those, since they allow to exert forces and produce tracking signals in a wireless fashion. In particular, we proposed to use implantable magnets as a HMI towards a new generation of myokinetic limb prostheses. If proven viable, the latter would entail parallel and independent control over multiple movements of an artificial hand, in a very natural and biomimetic fashion: hand prostheses with such capabilities are still a chimera. However to enable the implementation of this vision *in vivo*, magnet biocompatibility, stability and favorable integration within the host muscle tissue are essential conditions. In this paper we investigated three biocompatible materials as NdFeB magnet coatings and characterized their mechanical, corrosion and biological stability in comparison with general purpose uncoated magnets, generally recognized as not stable and poorly biocompatible. *In vitro* tests revealed the superiority of Par coatings compared to gold and titanium nitride, in terms of mechanical resistance and corrosion prevention when subjected to muscle-like stresses and post-surgery inflammatory environment for 30 days. This can be ascribed to material chemistry, mainly. In fact, magnets surface characterization revealed no significant difference among the three coatings in terms of defects or inhomogeneities. However, eventual differences in the 3D coating distribution or in coating thickness (that were not directly assessed in this study) may have influenced the performance of the magnets with different coatings in biological and mechanical stability studies, as well. Although TiN-coated magnets featured good mechanical and corrosion resistance in standard muscle environment, they failed in corrosion prevention when exposed to alkaline environments. *In vitro* biological tests performed with myoblasts in direct physical contact with the target specimen, revealed excellent performance of Par coated magnets. This proved true both in terms of metabolic activity and DNA content, with statistically significant differences with respect to uncoated magnets, and as shown by a high metabolic activity, well above the 70% of the negative control, in accordance with the reference standards.

Based on the results obtained *in vitro*, Par coated NdFeB magnets were chosen for implantation in rabbits, to evaluate their sub-acute toxicity and microscopic host response over 28 days. Acute systemic toxicity was evaluated as well through target specimen polar and non-polar extracts. *In vivo* validation revealed that the target specimen was locally non irritant and systemically non toxic. These results demonstrate the suitability of parylene C as coating material to stably adhere on the surface of NdFeB magnets and to prevent their corrosion and adverse host response upon implantation in muscle. This extends the range of possible applications of parylene C, with respect to the current state-of-the-art. In particular, it paves the way to the clinical translation of the myokinetic interface for limb prosthesis control, but also invites other possible biomedical applications in which the interaction between magnets and muscles is required.

## 4. Experimental section

### 4.1 Coated NdFeB permanent magnets

NdFeB disc-shaped permanent magnets (N52 grade, diameter: 3 mm, thickness: 1 mm, Magnet Sales & Service Limited, UK) covered by a NI–Cu–Ni layer and physically suitable for muscle implantation were used in this study and coated with biocompatible materials: Au, TiN and parylene C.

Au coatings were deposited through electroplating on metallic surfaces (*i.e.*, the NI–Cu–Ni layer) while TiN and Par coatings were deposited through Chemical Vapour Deposition (CVD) under vacuum to obtain relatively thick, stable, and well-adhered coatings. In all cases the deposition parameters were tuned to obtain a 10 μm-thick coating over the Ni–Cu–Ni (external) layer of the magnet.

### 4.2 Mechanical and corrosion resistance of the coatings

The mechanical stress applied on a magnet implanted in a muscle was simulated through compression cycles (15 000 cycles, frequency: 0.5 Hz). The number of cycles was chosen according to the number of grasps expected for a prosthetic hand in one month.^46^ The force applied on the magnet during the loading phase was ∼5 N. This value was fixed to reproduce on the sample surface the maximum intramuscular pressure expected^26,27^ in a worst-case scenario, thus considering a multiplicative factor equal to 10. Mechanical stress tests were performed on three magnets per each coating type using a dedicated setup including: (i) a motorized linear stage (VT-80 linear stages, PIS miCos GmbH), equipped with (ii) a load cell to monitor and control the compression force on the sample and (iii) a silicone disc (3 mm diameter, 1 mm thickness) acting as a substrate for the magnet. The silicon (E00-30 Ecoflex) was selected based on literature evidences to reproduce on the magnet the stress distribution and eventual damping effects that could be experience upon muscle implantation.^47^ The optical inspection of the samples was performed both through traditional microscopy and optical profilometry techniques. The former (KH-7700, Hirox) enabled to observe the entire magnet surface with a resolution of hundreds of micrometers. The latter (DCM 3D, Leica), enabled to detect eventual discontinuities and damages on the surface with a higher resolution when inspecting smaller surface portions.

Corrosion tests of the coatings in a muscle-mimicking environment, both in physiological and post-surgery inflammatory states were performed over 30 days by immersing the coated or uncoated magnets (positive control samples) in two PBS solutions (Sigma Aldrich) and by incubating them at 37 °C to reproduce the internal body temperature (thermostatic orbital oscillator – model 711/ct Elettrofor) with daily soaking medium replacement. The first solution consisted in simple PBS (pH 7.4) to simulate standard muscle conditions. The second was obtained by PBS acidification though HCl (37%, Sigma-Aldrich) and H_2_O_2_ (15 mM) to accomplish pH values of ∼5 (inflammatory state condition). H_2_O_2_ concentration was set based on literature data to reproduce the inflammatory cascade typically activated by the immune system to avoid tissue infection (which is known to produce ROS and pH values of ∼5). In particular, considering the concentration of H_2_O_2_*in vivo* (0.1 μM (ref. 48)) and the induced oxidative stress concentration (∼1 mM), we chose a concentration of H_2_O_2_ of 15 mM (ref. 49–52) (a worst-case scenario) and an inflammatory period of 4 weeks.^51,52^

Resistance to corrosion aimed to evaluate coating damages and eventual delamination was assessed through optical analysis on four magnets per each experimental condition, by considering 1, 7, 30 days as time-points.

### 4.3 *In vitro* biological evaluation: cell cultures and cytotoxicity assays

The C2C12 cell line (ATCC® CRL1772™, ATCC®, Manassas, Virginia, USA) was used. This is a subclone of the mouse myoblast cell line established by D. Yaffe and O. Saxel^53^ and was already used for *in vitro* evaluation of implantable muscular tissue scaffolds^54^ or bioeffects induced by physical therapies such as ultrasound.^55^ The biological response of the three magnet coatings and of the uncoated magnets (control condition) was evaluated both qualitatively using fluorescence and bright field microscopy analyses, and quantitatively using WST-1 cell metabolism assay and DNA content assessment with the PicoGreen kit. The data collected through WST-1 and PicoGreen assays were statistically evaluated using the Kruskal–Wallis post-hoc test with Dunn's multiple comparison test in order to identify significant differences among the coatings under investigation. Results were considered statistically different for *p*-values ≤0.05.

#### Cell culture and sample preparation

The cells were maintained at 37 °C and 5% CO_2_ in a growth medium composed by Dulbecco's modified Eagle's medium (DMEM, Sigma-Aldrich), supplemented with 10% fetal bovine serum and 1% penicillin-streptomycin (PEN/STREP, Gibco). After reaching 80% confluence, the cells were split 1 : 3 and maintained for 48 h in T-flasks. Then, the cells were harvested with trypsin/ETDA (0.5 mg mL^−1^, Gibco) and seeded with a density of 10 000 cell per cm^2^ in 96-well plates in direct contact with the magnets. Two time points were considered (24 and 72 hours upon seeding) and eight samples of the three coating materials were employed per each time point. Uncoated magnets (Ni-magnets) were used as a positive control, whereas C2C12 cells seeded on polystyrene wells (in the absence of any magnet) as a negative control. Before cell seeding, the magnets were washed with acetone for 5 minutes. Sample sterilization was performed by cleaning with 70% ethanol for 30 min, four cycles of sterile water washing, and 30 minutes UV-light exposure under a sterile hood.

#### Cell imaging

Imaging of cells was performed after 24 h and 72 h from seeding, both in bright field and fluorescence. In the latter case, cells were incubated with a 2 μM calcein AM (LIVE/DEAD Viability/Cytotoxicity Kit, Invitrogen Corporation) and 1 μM Hoechst (Thermo Fisher Scientific) solution in PBS (concentration 1 : 1000), to assess intracellular esterase activity/viability and to stain the cellular nuclei, respectively. The staining solution was added to each well, followed by incubation at 37 °C for 10 min. Images of the blue channel (nuclei, bandpass filter value equal to 461 nm) and green channel (cytosol of viable cells, bandpass filter value equal to 517 nm) were acquired at a magnification of 10× with an optical microscope (ECLIPSE Ti-E Inverted epifluorescence microscope, Nikon Instruments).

#### WST-1 assay

C2C12 cells metabolic activity after 24 h and 72 h incubation with magnetic samples was assessed through WST-1 Cell Proliferation Colorimetric Reagent test kit (based on the 2-(4-iodophenyl)-3-(4-nitophenyl)-5-(2,4-disulfophenyl)-2*H*-tetrazolium monosodium salt, provided in a pre-mix electro-coupling solution, BioVision). This method is based on the reduction of tetrazolium salt by a mitochondrial dehydrogenase to produce formazan dye, which content is therefore proportional to the amount of metabolically intact cells. Cultures were incubated with a 1 : 11 dilution of WST-1 reagent in growth medium for 1 h at 37 °C with 5% CO_2_, according to the manufacturer protocol. The dye-containing supernatant was then transferred to a new 96-well plate to perform spectrophotometer analysis (absorbance measures at 450 nm, performed with a microplate reader Victor 3, PerkinElmer).

#### PicoGreen assay

The Quant-iT PicoGreen assay (Thermo Fisher Scientific) was performed on the same cell samples used for the WST-1 assay, being the latter a non destructive assay. Briefly, after supernatant removal and PBS washing, distilled water was added to the samples. Three freeze–thaw cycles were executed to lyse cell membrane physically. The lysates were then treated according to the kit manufacturer's instructions. The fluorescence intensity (proportional to the DNA content) was measured using the microplate reader with an excitation wavelength of 485 nm and an emission wavelength of 535 nm. This measure enabled to relate the cell metabolic activity to the DNA content of each well.

### 4.4 *In vivo* biological evaluation

All animal experiments were performed in an Association for Assessment and Accreditation of Laboratory Animal Care (AAALAC) accredited facility in accordance with the recommendation of the Committee for the Purpose of Control and Supervision of Experiments on animals (CPCSEA) guidelines for laboratory animal facility (India, 2018) and in accordance with the International Standard ISO 10993. Experimental protocols were approved by the Institutional Animal Ethics Committee (IAEC) (Protocol No. BIO-IAEC-3679, BIO-IAEC-3651, BIO-IAEC-3603).

#### Acute systemic toxicity tests

0.9% w/v sodium chloride and sesame oil were selected as polar and non-polar solvents, respectively for extracts preparation (according to ISO 10993). Polar and non-polar extracts were prepared from sterilized Par coated magnets at a 0.2 g mL^−1^ extraction ratio (extraction time 72 h, 50 °C). After verifying the absence of any suspended particle, the extracts were stored at room temperature and injected within 24 h after completion of the extraction. 20 female 8–9 weeks old Swiss albino mice weighing between 22.15 and 24.15 g were randomly divided into four groups (G1 = polar solvent, G2 = magnet extracts in polar solvents, G3 = non-polar solvent, G4 = magnet extracts in non-polar solvents). Polar and non-polar solvents were injected though the intravenous and intraperitoneal routes, respectively, in a dose of 50 mL kg^−1^. All the animals were observed for clinical signs of toxicity at seven time points upon extracts injection (30 min, 1-2-4-48-72-96 h).

#### Pirogenicity tests

6 male 4 months-old New Zeland white rabbits weighing between 1.58 and 2.72 kg were randomly divided in two groups, namely a control and a test group. The control group was administered with blank extracts (saline solution undergoing the same thermal treatment as the sample extracts), whereas the test group with the Par coated magnet extract.

Magnet extracts were prepared from sterilized Par coated magnets at a 0.2 g mL^−1^ extraction ratio (extraction time 72 h, 50 °C) in 0.9% NaCl solution under continuous mechanical shaking. The extracts were stored at room temperature and injected within 24 h after completion of the extraction. The absence of any suspended particle was verified prior to injection. Extracts were pre-heated at 37.2 °C before intravenous injection through a marginal ear vein at a dose volume of 10 mL kg^−1^. Temperature was monitored both through a digital thermometer (for baseline temperature recording) and temperature sensor probes inserted in the rabbit rectum. Baseline temperature was recorded 30 min before the injection and rectal temperature was recorded every 30 min for 3 h post-injection.

#### Implantation tests

Implantation tests were performed to assess local effects and systemic toxicity. Host response induced by Par coated magnets was compared with that of negative control samples (high-density polyethylene film, HDPE-RM-C) upon intramuscular implantation in 4 months-old New Zeland white rabbits weighing between 2.39 and 2.49 kg. Rabbits were randomly divided into three groups (GR1 – GR3). Two follow-up periods were considered, namely 7 (GR3) and 28 days (GR1, GR2) upon implant. GR1 and GR2 received bilateral paravertebral intramuscular implants of either control samples (GR1) or coated magnets (GR2). GR3 was implanted both with Par coated magnets and control samples.

HDPE-RM-C was properly shaped to obtain 2 × 10 mm strips without any sharp edge to avoid mechanical trauma. HDPE samples and coated magnets were sterilized prior to implantation. Anesthesia was induced by intramuscular injection with a combination of Xylazine and Ketamine at the dose of 5 and 35 mg kg^−1^, respectively. The dorsal and dorso-lateral sides of the rabbits were shaved and disinfected with 5% w/v povidone-iodide solution and let dry before the surgery. A surgical incision was made on each side of the back parallel to the lumbar region of the spine. The skin incision was extended to paravertebral muscles. After sterile samples inclusion, absorbable and non-absorbable suture wires were employed to close muscle and skin incision, respectively. The animals were observed daily to detect signs of toxicity, to measure body weight and to monitor food consumption. At either 7 or 28 days post-implantation, the animals were euthanized by sodium thiopentone intravenous administration. Hematology and urine analyses were performed for GR1 and GR2 before sacrifice. To enable histopathological examination, organs from all the euthanized animals were preserved in 10% w/v Neutral Buffered Formalin and Modified Davidson's fixative for optical observation. Later the implant site with surrounding tissue, were embedded in paraffin wax, sectioned at 4–5 μm (three sections per animal) and stained with hematoxylin and eosin. In accordance to ISO 10993-6:2016(E) a score for the implantation site was attributed based on the extent of the fibrotic capsule, inflammation state, eventual presence of necrosis, *etc.*

The stained sections were visualized through light microscopy (Axio Scope A1, Zeiss). Three randomly identified high magnification field images were examined (5× objective lenses for photography and 5×/10×/40× for evaluation, each field 1200 × 900 μm). For microscopic evaluation, implant sites were scored based on the inflammatory cells that migrated to the site (number per high powered field and width of zone surrounding the implant), presence of necrosis, fibrosis, vascularization, fat infiltration and other tissue alterations. The reaction for the control material was subtracted from the reaction of the test material. The resultant score was correlated to the control sample and an irritation score was obtained.

A semi-quantitative scale was used to assess the infiltration of lymphocytes, macrophages, and neovascularization based on the scoring system defined by ISO 10993–6 (2016) (Table 1).

## Conflicts of interest

There are no conflicts to declare.

## Supplementary Material

RA-011-D0RA07989H-s001
