# Supplementary material for: Stability and in vivo safety of gold, titanium nitride and parylene C coatings on NdFeB magnets implanted in muscles towards a new generation of myokinetic prosthetic limbs
Source: RSC Adv. 2021 Feb 8;11(12):6766–75. doi: 10.1039/d0ra07989h (PMC8694929; doi:10.1039/d0ra07989h)
Supplement: RA-011-D0RA07989H-s001 [file RA-011-D0RA07989H-s001.pdf]

Electronic Supplementary Material (ESI) for RSC Advances.
This journal is © The Royal Society of Chemistry 2021

Supplementary Materials

Title:

Stability and in vivo safety of gold, titanium nitride and parylene C coatings on NdFeB magnets implanted in muscles towards a new generation of myokinetic prosthetic limbs

Veronica Iacovacci[a,b], Irene Naselli[a], Alice Rita Salgarella[a], Francesco Clemente[a,b], Leonardo Ricotti[a,b], and Christian Cipriani[a,b]

a. The BioRobotics Institute, Scuola Superiore Sant'Anna, Piazza Martiri della Libertà 33, 56127 Pisa, Italy

b. Department of Excellence in Robotics & AI, Scuola Superiore Sant'Anna, Piazza Martiri della Libertà 33, 56127 Pisa, Italy

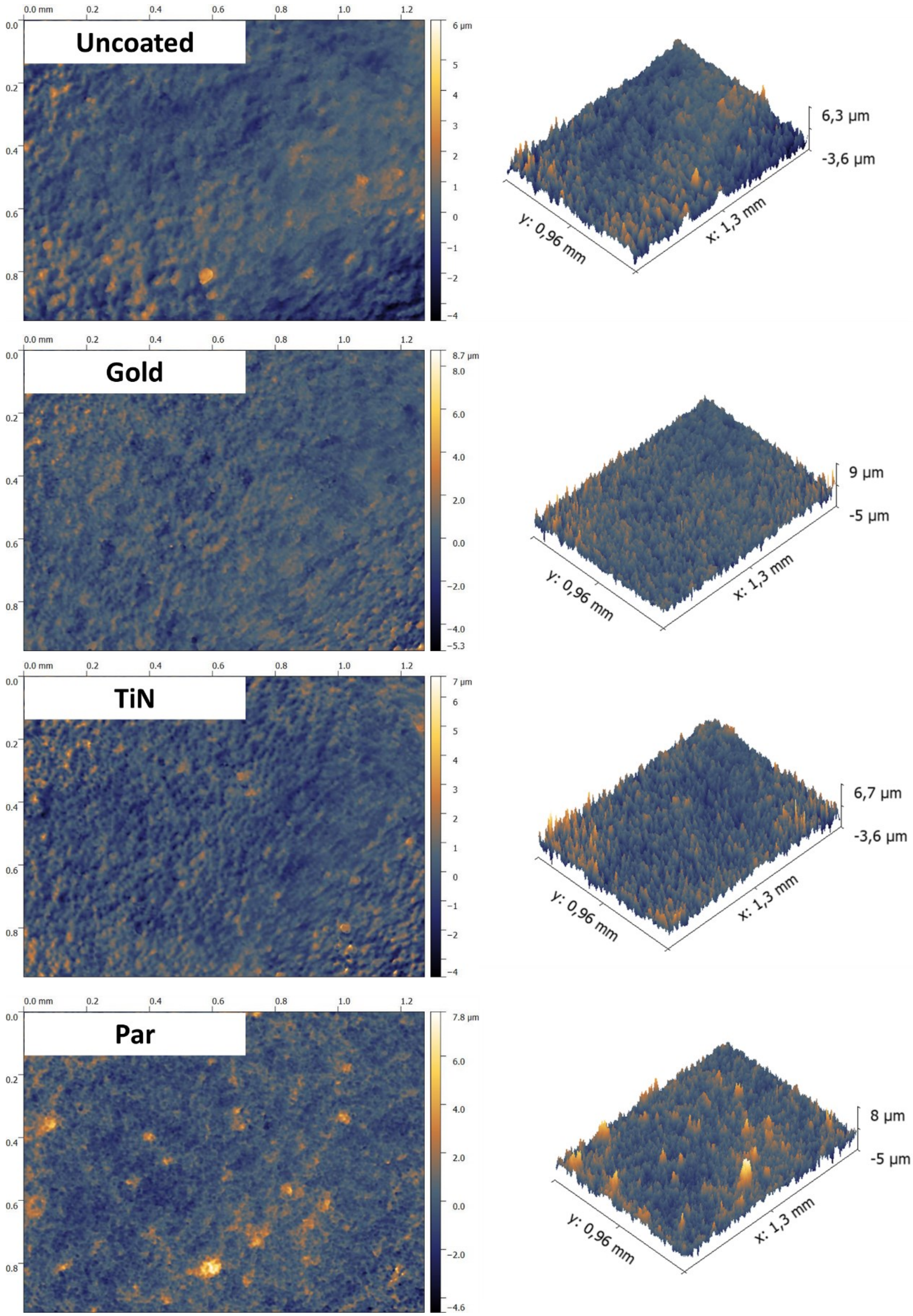

Fig. S 1: Surface characterization through optical profilometer of Uncoated and coated magnets (Gold, TiN, Par). Both 2D and 3D surface scansions are reported for each material. Optical profilometer analysis (DCM 3D, Leica, Germany), relying on the combination of confocal microscopy and interferometry techniques, was used to quantitatively assess surface roughness. Samples with different coatings were scanned (1.27 x 0.95 mm2 scanned area, three samples per coating type and three images in different locations for each sample) and three dimensional surface profiles were acquired and processed through the Gwyddion software.

Table S 1: Roughness parameters derived from optical profilometer analysis. Ra is the arithmetic average height; Rq is the rooth mean square error roughness; Rsk is the skewness; Rp is the maximum height of peak; Rv is the maximum depth of valleys.

	Ra [µm]	Rq [µm]	Rsk	Rp [µm]	Rv [µm]
**Uncoated**	0.52±0.08	0.69±0.11	0.06±0.43	5.34±0.77	-3.65±1.43
**Gold**	0.64±0.03	0.83±0.04	0.03±0.16	8.93±3.45	-5.07±0.88
**TiN**	0.60±0.03	0.79±0.03	0.45±0.11	7.28±1.67	-3.90±1.18
**Par**	0.74±0.09	0.99±0.11	0.85±0.39	6.87±1.29	-4.91±1.01

Table S2: Clinical signs of toxicity and mortality record in acute systemic toxicity studies (N stands for Normal).

Group, sex and group description	Animal No.	Clinical signs of Toxicity/Mortality						
		Observation on Day 1				Day		
		30 min	1 h	2 h	4 h	2	3	4
G1, Female, Polar solvent control	1	N	N	N	N	N	N	N
	2	N	N	N	N	N	N	N
	3	N	N	N	N	N	N	N
	4	N	N	N	N	N	N	N
G2, Female, Polar Test Item extract	5	N	N	N	N	N	N	N
	6	N	N	N	N	N	N	N
	7	N	N	N	N	N	N	N
	8	N	N	N	N	N	N	N
G3, Female, Non-Polar solvent control	9	N	N	N	N	N	N	N
	10	N	N	N	N	N	N	N
	11	N	N	N	N	N	N	N
	12	N	N	N	N	N	N	N
G4, Female, Non-Polar Test Item extract	13	N	N	N	N	N	N	N
	14	N	N	N	N	N	N	N
	15	N	N	N	N	N	N	N
	16	N	N	N	N	N	N	N

Table S3: Administered extract dose and body weight changes in acute systemic toxicity studies.

Group, sex and	Animal	Administered	Body weight (g) on Days	Percentage of weight change

group description	No	Dose (mL)					with respect to Day		
			1	2	3	4	1-2	1-3	1-4
G1, Female, Polar solvent control	1	1.2	23.14	23.25	23.37	23.49	0.48%	0.99%	1.51%
	2	1.2	23.73	23.85	23.98	24.09	0.51%	1.05%	1.52%
	3	1.2	24.01	24.12	24.25	24.37	0.46%	1.00%	1.50%
	4	1.2	24.63	24.75	24.86	24.98	0.49%	0.93%	1.42%
	5	1.2	24.82	24.93	25.04	25.17	0.44%	0.89%	1.41%
	**Mean**		**24.07**	**24.18**	**24.30**	**24.42**	**0.47%**	**0.97%**	**1.47%**
	**±SD**		**0.68**	**0.68**	**0.68**	**0.68**	**0.02%**	**0.06%**	**0.05%**
G2, Female, Polar Test Item extract	6	1.2	23.24	23.36	23.47	23.59	0.52%	0.99%	1.51%
	7	1.2	23.41	23.53	23.65	23.77	0.51%	1.03%	1.54%
	8	1.2	24.10	24.22	24.35	24.48	0.50%	1.04%	1.58%
	9	1.2	24.74	24.86	24.97	25.10	0.49%	0.93%	1.46%
	10	1.2	24.82	24.93	25.05	25.27	0.44%	0.93%	1.81%
	**Mean**		**24.06**	**24.18**	**24.30**	**24.44**	**0.49%**	**0.98%**	**1.58%**
	**±SD**		**0.73**	**0.73**	**0.73**	**0.76**	**0.03%**	**0.05%**	**0.14%**
G3 Female, Non-Polar solvent control	11	1.2	23.24	23.35	23.47	23.59	0.47%	0.99%	1.51%
	12	1.2	23.58	23.70	23.82	23.95	0.51%	1.02%	1.57%
	13	1.2	23.86	23.98	24.11	24.23	0.50%	1.05%	1.55%
	14	1.2	24.77	24.89	24.01	24.14	0.48%	-3.07%	2.54%
	15	1.2	24.89	24.96	25.08	25.19	0.28%	0.76%	1.21%
	**Mean**		**24.07**	**24.18**	**24.10**	**24.22**	**0.45%**	**0.15%**	**1.68%**
	**±SD**		**0.73**	**0.72**	**0.60**	**0.60**	**0.10%**	**1.80%**	**0.50%**
G4, Female, Non-Polar Test Item extract	16	1.2	23.35	23.45	23.57	23.69	0.43%	0.94%	1.46%
	17	1.2	23.69	23.82	23.95	24.07	0.55%	1.10%	1.60%
	18	1.2	23.99	24.11	24.19	24.31	0.50%	0.83%	1.33%
	19	1.2	24.50	24.62	24.73	24.85	0.49%	0.94%	1.43%
	20	1.2	24.81	24.93	25.05	25.17	0.48%	0.97%	1.45%
	**Mean**		**24.07**	**24.19**	**24.30**	**24.42**	**0.49%**	**0.96%**	**1.45%**
	**±SD**		**0.59**	**0.60**	**0.59**	**0.59**	**0.04%**	**0.09%**	**0.10%**

Table S4: Gross pathology findings in acute systemic toxicity studies (TS:Terminal Sacrifice, NAD: No Abnormality Detected)

Group, sex and group description	Animal No.	Fate	Gross Pathology

			External	Internal
G1, Female, Polar solvent control	1	TS	NAD	NAD
	2	TS	NAD	NAD
	3	TS	NAD	NAD
	4	TS	NAD	NAD
	5	TS	NAD	NAD
G2, Female, Polar Test Item extract	6	TS	NAD	NAD
	7	TS	NAD	NAD
	8	TS	NAD	NAD
	9	TS	NAD	NAD
	10	TS	NAD	NAD
G3, Female, Non-Polar solvent control	11	TS	NAD	NAD
	12	TS	NAD	NAD
	13	TS	NAD	NAD
	14	TS	NAD	NAD
	15	TS	NAD	NAD
G4, Female, Non-Polar Test Item extract	16	TS	NAD	NAD
	17	TS	NAD	NAD
	18	TS	NAD	NAD
	19	TS	NAD	NAD
	20	TS	NAD	NAD

Table S5: Pirogenicity test: temperature measurements.

	Animal No.	Body weight (kg)	Dose (mL)	Temperature				
				Control Temperature	After administration			
					60 min	90 min	120 min	150 min
**Blank extract**	1	1.85	18.5	37.8	37.8	37.9	38.0	38.0
	2	2.21	22.1	38.4	38.5	38.5	38.5	38.0
	3	2.38	23.8	38.3	38.4	38.7	38.7	38.0
**Test Item extract**	4	1.97	19.7	38.2	38.5	38.5	38.5	38.4
	5	1.66	16.6	38.8	38.8	38.8	38.7	38.6
	6	1.61	16.1	38.5	38.6	38.7	38.7	38.7

Table S6: Individual animal hematological records.

**Group, sex and group description**	**Animal No**	**Total leucocyte count (x 10^3 cells/µL)**	**Total erythrocyte count (x 10^6 cells/µL**	**Hemoglobin (g/dL)**	**Hematocrit (%)**	**Platelet count (x 10^3 cells/µL)**	**Reticulocyte count (%)**	**Neutrophils (%)**	**Lymphocytes (%)**
G_R1, M, Negative Control	1	8.70	6.72	13.5	41.1	727	1.95	22.8	66.9
	2	9.49	5.36	11.1	36.4	561	7.89	22.7	68.5
	3	9.16	5.47	114.4	37.2	531	8.11	24.7	67.5
G_R2, M, Test Item	4	8.46	5.60	12.0	38.1	666	3.76	40.4	48.0
	5	11.61	6.04	12.9	39.9	549	2.63	56.1	32.1
	6	5.73	6.29	13.2	43.1	549	4.58	41.3	43.0
G_R1, F, Negative Control	7	12.22	5.17	10.6	34.2	607	4.94	39.3	35.2
	8	8.27	4.89	10.4	32.5	755	4.94	37.6	52.5
	9	8.83	6.07	13.2	41.8	494	3.91	26.4	60.7
G_R2, F, Test Item	10	17.94	4.68	9.4	32.8	950	3.77	59.0	27.8
	11	18.68	4.65	9.5	32.5	1008	3.48	55.3	32.3
	12	5.65	5.07	10.6	35.5	302	7.29	26.3	49.3

Table S 7 Individual animal urinalysis record

Group, sex and Group description	Animal No	Urobilinogen (mg/dL)	Bilirubin (mg/dL)	Ketones (mg/dL)	Blood (RBC/µL)	Protein (mg/dL)	Nitrite	Leucocytes (LEU/µL)	Glucose (mg/dL)	pH	Microalbumin (mg/dL)
G_R1, M, Negative Control	R1	0.2	Neg	Neg	Neg	Neg	Neg	Neg	Neg	7.5	15
	R2	0.2	Neg	Neg	Neg	Neg	Neg	Neg	Neg	7.0	15
	R3	0.2	Neg	Neg	Neg	Neg	Neg	Neg	Neg	7.5	15
G_R2, M, Test Item	R4	0.2	Neg	Neg	Neg	Neg	Neg	Neg	Neg	7.5	15
	R5	1	1	Neg	Neg	100	Neg	Neg	Neg	8.5	>15
	R6	0.2	Neg	Neg	Ca10	Neg	Neg	Neg	Neg	6.0	15
G_R1, F, Negative Control	R7	0.2	Neg	5	Neg	Trace	Neg	Neg	Neg	5.5	>15
	R8	0.2	Neg	Neg	Neg	Neg	Neg	Neg	Neg	8.5	15
	R9	1	Neg	Neg	Ca80	30	Neg	Ca70	Neg	8.5	>15
G_R2, F, Test Item	R10	0.2	Neg	Neg	Neg	Neg	Neg	Neg	Neg	6.5	15
	R11	0.2	Neg	Neg	Neg	Trace	Neg	Neg	Neg	7.0	>15
	R12	0.2	Neg	5	≥Ca200	Neg	Neg	Neg	Neg	8.0	15
